# Understanding obesity–cancer crosstalk to inform the immunomodulatory roles of anti-obesity nanotherapeutics

**DOI:** 10.1016/j.mtbio.2025.102756

**Published:** 2025-12-31

**Authors:** Fang Zhou, Xiaofang Li, Jianping Jiang, Lingxiao Zhang

**Affiliations:** a Traditional Chinese Medical Hospital of Zhuji, Shaoxing 311800, China; b School of Medicine, Hangzhou City University, Hangzhou 310015, China; c Interdisciplinary Nanoscience Center, Aarhus University, Aarhus C, 8000, Denmark

**Keywords:** Obesity, Cancer, Adipose inflammation, Macrophages, Anti-inflammation

## Abstract

Obesity represents a chronic inflammatory condition characterized by adipose tissue dysfunction that drives systemic metabolic derangements and promotes tumor progression. Adipose inflammation, dominated by pro-inflammatory macrophages and dysregulated adipokine secretion, not only impairs lipid metabolism but also suppresses anti-tumor immunity. Although anti-obesity medications (AOMs) can induce weight loss, their limited ability to resolve inflammation constrains long-term metabolic and oncologic benefits. Recent advances in nanomedicine have enabled targeted modulation of adipose tissue through enhanced drug bioavailability, controlled release, and remodeling of the immune microenvironment. This review summarizes recent progress in anti-inflammatory nanomedicines for obesity and their potential to synergize with cancer therapy. Specifically, we discuss nanomedicines that alleviate adipose inflammation *via* macrophage reprogramming, cytokine silencing, or reactive oxygen species scavenging, as well as those that promote white adipose tissue browning and brown adipose tissue activation and thermogenesis. By elucidating the interplay between adipose inflammation, metabolic regulation, and cancer immunotherapy, we propose that natural compounds and drug-free nanomaterials targeting adipose inflammation may serve as a critical foundation for next-generation nanotherapeutics, offering a dual-benefit strategy to combat obesity and enhance cancer immunotherapy efficacy.

## Graphical abstract

**Figure:**
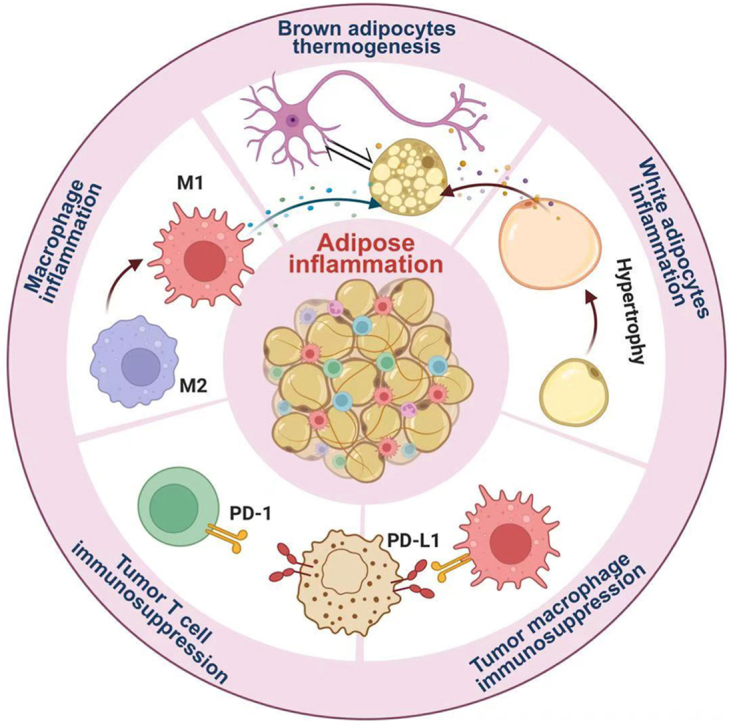

## 1 Introduction

Obesity, defined as a body mass index (BMI) > 30 kg/m^2^, has become a major global public health challenge, affecting over 16 % of adults and 8 % of children and adolescents worldwide [1,2]. Alarmingly, the prevalence is projected to rise to nearly 60 % of adults and 31 % of children by 2050 [3]. Obesity primarily results from an imbalance between caloric intake and energy expenditure [4]. Excess energy is stored in white adipose tissue (WAT), which is distributed mainly in subcutaneous adipose tissue (SAT) and visceral adipose tissue (VAT) compartments. Generally, VAT hypertrophy (enlargement of existing adipocytes) and SAT hyperplasia (formation of new adipocytes through differentiation of preadipocytes) represent the two primary pathways of fat storage [5]. Hypertrophic VAT adipocytes, however, are prone to cell death and inflammatory processes [6]. Mechanistically, as adipocytes expand, they encounter mechanical stress from increased contact with neighboring cells and the extracellular matrix, as well as hypoxia due to limited oxygen diffusion. Both stresses promote adipose tissue inflammation [7,8]. Moreover, hypertrophic adipocytes acquire altered biochemical properties, including elevated lipolysis, increased secretion of pro-inflammatory cytokines such as tumour necrosis factor (TNF) and interleukin 6 (IL-6), and reduced secretion of anti-inflammatory adipokines such as leptin and adiponectin [[9], [10], [11]]. Adipose inflammation, a result of VAT Hypertrophy, drives a state of chronic low-grade inflammation, ultimately leading to lipotoxicity, systemic inflammation, and metabolic syndromes [12].

Obesity is strongly associated with a wide range of comorbidities, including type 2 diabetes and cardiovascular diseases [13], several cancers [14,15], neurological disorders [16] and chronic conditions such as non-alcoholic fatty liver disease (NAFLD) [17,18]. Sustained weight loss of more than 10 % of body weight can substantially improve many obesity-related complications while enhancing quality of life [19]. However, current treatment approaches have limited long-term effectiveness. Behavioral interventions are often followed by weight regain [20], physical exercise alone typically produces only modest weight reduction [21], and bariatric surgery, while effective, is constrained by high cost and surgical risk [22]. To address these limitations, several anti-obesity medications (AOMs) have been approved by the U.S. Food and Drug Administration (FDA) [23]. Evidence from 56 clinical trials involving these AOMs, including orlistat (22), semaglutide (14), liraglutide (11), tirzepatide (6), naltrexone/bupropion (5), and phentermine–topiramate (2), demonstrated that all agents achieved significantly greater body weight reduction compared with placebo (*P* < 0.0001), with semaglutide and tirzepatide achieving >10 % total body weight loss [24]. Nevertheless, the clinical management of obesity remains challenging, as AOMs are often limited by insufficient efficacy, safety concerns, and long-term tolerability issues (Table 1) [25].

**Table 1:** The usage and side effects of AOMs approved by FDA.

Medications	Dosage	Weight loss^a^	Common side effects
Semaglutide	0.25–2.4 mg/wk, subcutaneously	7 %–15 %	Nausea, diarrhea, constipation, dyspepsia, vomiting
Liraglutide	0.6–3 mg/d, subcutaneously	5 %–10 %	Nausea, diarrhea, constipation, dyspepsia, vomiting
Tirzepatide	2.5–15 mg/wk, subcutaneously	15 %–21 %	Nausea, diarrhea, vomiting, constipation, decreased appetite, abdominal pain
Phentermine-topiramate	3.75/23 mg/d to 15/92 mg/d, orally	7 %–9 %	Hypertension, tachycardia, palpitations, insomnia, anxiety, diarrhea, constipation, nephrolithiasis
Naltrexone-bupropion	8 mg/90 mg daily to 16 mg/180 mg twice daily, orally	5 %–10 %	Abdominal pain, vomiting, constipation, dry mouth, headaches, tinnitus, vertigo, dysgeusia, insomnia
Orlistat	60–120 mg 3 times daily, orally	4 %–10 %	Steatorrhea, increased defecation, oily spotting, liquid stool, faecal spotting

a Data was calculated in 24 months.

Chronic low-grade inflammation induced by obesity is a central mechanism linking obesity to cancer development. Epidemiological studies report that obesity significantly increases the risk of mortality from cancers of the esophagus, colorectum, liver, gallbladder, pancreas, and kidney [[26], [27], [28]]. It is estimated that 4–9 % of all cancer diagnoses are attributable to excess body fat, and obesity is consistently associated with poorer prognosis across multiple malignancies [29,30]. Mechanistically, obesity not only drives pathological inflammation but also impairs anti-tumor immune responses, thereby fostering a pro-tumorigenic environment [31]. Furthermore, patients with overweight or obesity and concomitant low muscle mass experience higher rates of surgical complications, treatment-related toxicities, recurrence, and cancer-specific mortality compared with patients of normal BMI [31]. The development of anti-obesity medications (AOMs) and emerging nanomedicines for weight loss and obesity management has been extensively reviewed in recent literature [25,32]. However, these studies have not clarified how AOMs modulate adipose tissue inflammation or the potential anti-cancer benefits associated with this process. Distinguishing this review from prior works, we integrate mechanistic insights from adipose immunometabolism with cancer immunology to elucidate how dysfunctional adipose inflammation suppresses systemic and tumor immunity. Beyond describing the pathology, we analyze nanomedicine strategies that specifically target these adipose inflammatory pathways, ultimately framing adipose tissue as an overlooked yet therapeutically actionable immune organ in cancer.

## 2 Adipose inflammation

**Fig. 1:**
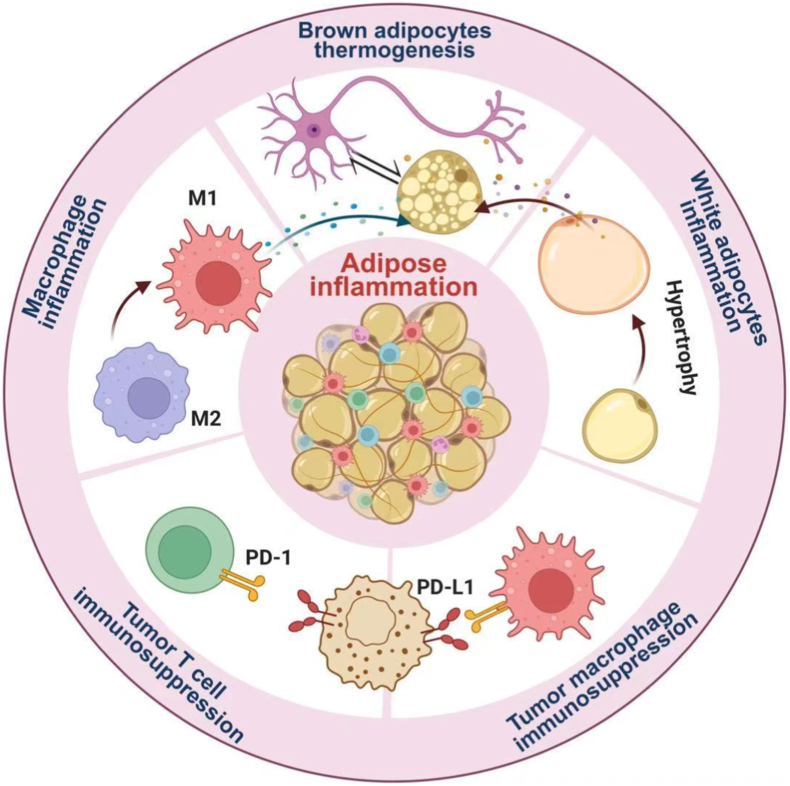
**Schematic illustration of how adipose inflammation affects obesity and cancer therapy.** Created in BioRender (2025).

WAT hypertrophy and pro-inflammatory polarization of adipose tissue macrophages play pivotal roles in driving local adipose inflammation and systemic chronic low-grade inflammation. The resultant inflammatory microenvironment not only impairs brown adipose tissue (BAT) activity and thermogenesis but also enhances the expression of the immune checkpoint PD-1 in tumor-associated macrophages and effector T cells (Fig. 1). Together, these processes establish a pathological link between obesity-associated inflammation and tumor immune suppression. In the following section, we briefly discuss how adipose inflammation influences both obesity progression and cancer therapy outcomes.

### 2.1 Adipose macrophages (ATMs) play a central role in adipose inflammation

Adipose tissue is composed of mature adipocytes, preadipocytes, and a diverse array of other cell types, including immune cells (*e.g.*, macrophages, neutrophils, lymphocytes), mesenchymal cells, and endothelial cells [33]. The initiation of adipose inflammation can be triggered by gut-derived substances, dietary components, or metabolites, as well as intrinsic signals arising during rapid adipose tissue expansion. These intrinsic signals include adipocyte death, hypoxia, and mechano-transduction from interactions between adipocytes and the extracellular matrix [34]. In response, adipocytes secrete biologically active adipokines, such as TNF, which promote the recruitment and migration of immune cells into adipose tissue and drive further adipogenesis [35]. A hallmark of adipose inflammation is the increased accumulation of pro-inflammatory (M1-polarized) macrophages, which secrete cytokines such as TNF and IL-1β [36,37]. In lean individuals, macrophages comprise approximately 5 % of adipose tissue cells, whereas in obesity they can constitute up to 40–50 % [38]. In turn, higher proportion of pro-inflammatory macrophages in the adipose tissue further contribute to a self-sustaining pro-inflammatory environment in obese individuals [39]. Mechanistically, HFD feeding promotes epidermal growth factor receptor signaling in adipose tissue macrophages, enhancing their proliferation and monocyte infiltration [40]. In parallel, adipose inflammation has been linked to metabolic reprogramming, including glutaminolysis-driven suppression of AMPK, activation of succinyl-CoA synthetase, and overproduction of succinate and IL-1β [41]. Crosstalk between inflammatory macrophages and adipose stem cells (ASCs) further exacerbates tissue dysfunction. Specifically, loss of TNF-α–induced protein 8-like 2 in visceral ATMs drives mitochondrial fragmentation and disrupts ferritin-containing exosome transfer to ASCs, leading to ROS and Fe^2+^ overload, ASCs ferroptosis, and aggravated HFD-induced obesity and metabolic dysfunction in male mice [42].

### 2.2 Current AOMs failed to address macrophage-mediated adipose inflammation

**Fig. 2:**
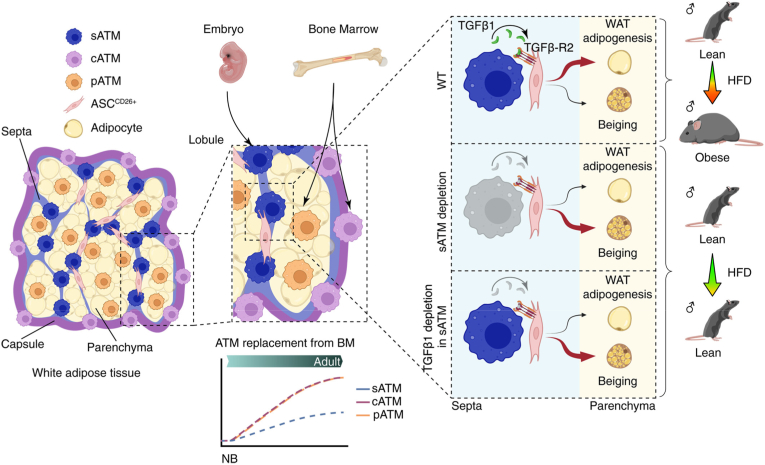
**Distinct ATMs regulate adipocyte formation.** WAT is divided into three structural regions—the septum, capsule, and parenchyma—each harboring unique subsets of ATMs. Within the septal zone, septal ATMs (sATMs) reside adjacent to early adipose progenitors marked by CD26 (ASCs^CD26+^). Their proximity allows sATMs to influence progenitor commitment through TGFβ1-mediated cues, thereby driving the development of lipid-storing white adipocytes [43]. Reproduced with permission from Copyright © 2025 the authors.

Notably, a study by Yu et al. identified a macrophage–progenitor axis within the septa that regulates adipose plasticity and systemic metabolism, highlighting septal ATMs as potential therapeutic targets for promoting WAT browning and countering metabolic disease (Fig. 2) [43]. They revealed that WAT contains three anatomically distinct macrophage subsets—parenchymal, capsular, and septal—with septal ATMs (CD209b^+^LYVE1^+^) forming a unique niche adjacent to early CD26^+^ adipocyte progenitors. These embryonically derived septal ATMs remained spatially confined and resistant to monocyte replacement during high-fat diet stress, unlike parenchymal ATMs, which expanded through proliferation and recruitment. Functionally, septal ATMs supplied TGFβ1 signals that directed progenitors toward white adipocyte differentiation and restrained thermogenic potential. Selective depletion of septal ATMs enhanced beige fat formation, increased energy expenditure, conferred resistance to diet-induced obesity, and improved glucose and insulin responses.

Despite advances in AOMs, these therapies remain largely ineffective in directly resolving macrophage-driven chronic inflammation [44]. Importantly, the effects of AOMs on adipose inflammation appear heterogeneous. Using a spatially resolved single-nucleus atlas of cells from 70 individuals, Miranda et al. characterized the cellular, molecular, and regulatory changes that reshape human adipose tissue and metabolic health in obesity and therapeutic weight loss. Their findings revealed that weight loss reduces adipocyte hypertrophy and relieves biomechanical stress, thereby enhancing global metabolic flux and bioenergetic substrate cycling to improve systemic metabolism. However, within the immune compartment, weight loss-mediated by semaglutide only partially repressed obesity-induced macrophage infiltration and did not fully reverse macrophage activation, leaving these cells primed for reactivation and thus predisposing individuals to weight regain and worsened metabolic dysfunction [45]. By contrast, preclinical studies demonstrated that tirzepatide effectively alleviates VAT inflammation in obese mice by inhibiting M1 macrophage infiltration, promoting apoptosis of pro-inflammatory macrophages, and reducing cytokine secretion through suppression of ERK phosphorylation [46]. Collectively, these findings highlight that while certain AOMs can modulate macrophage-driven inflammation, their therapeutic potential in this context remains to be fully established.

### 2.3 ATMs-mediated adipose inflammation hampers fat metabolism

Adipose tissue can be broadly classified into WAT, beige adipose tissue, and BAT. Unlike WAT, which primarily stores lipids, BAT is a major site of non-shivering thermogenesis in mammals and has long been considered an attractive therapeutic target for promoting weight loss [47]. BAT-derived heat is critical for survival in cold environments and for arousal in hibernating species. Brown adipocytes are enriched with mitochondria that express uncoupling protein-1 (UCP1), which uncouples oxidative phosphorylation by dissipating the proton gradient across the mitochondrial membrane, thereby stimulating respiratory chain activity and generating heat [48]. This heat is distributed systemically *via* circulation [49]. In addition to classical BAT, clusters of UCP1-expressing beige adipocytes can emerge within WAT in response to environmental or pharmacological stimuli. Similar to brown adipocytes, beige adipocytes are characterized by multilocular lipid droplets, high mitochondrial content, and expression of brown fat–specific genes such as UCP1, contributing to thermogenesis [50]. Obesity is associated with reduced BAT activity and impaired WAT browning, which contribute to metabolic dysfunction. Chronic adipose inflammation is now recognized as a key mechanism underlying BAT dysfunction in obesity [51]. Pellegrinelli et al. showed that high-fat diet (HFD) feeding progressively impairs diet-induced thermogenesis in parallel with the development of fibro-inflammation in BAT [52]. In this study, Pepd-heterozygous mice, which exhibit defective collagen turnover, display aggravated BAT dysfunction and fibro-inflammation under HFD and thermoneutral conditions. In humans, elevated fibro-inflammation markers correlate with reduced cold-induced BAT activity, and BAT inactivation under thermoneutrality also exhibits fibro-inflammatory features.

ATMs play a pivotal role in regulating BAT and beige adipocyte activity. Pro-inflammatory macrophages suppress thermogenesis by secreting cytokines that inhibit noradrenergic signaling and by degrading norepinephrine, thereby reducing sympathetic tone and thermogenic capacity. In contrast, anti-inflammatory macrophages promote WAT browning and BAT activity [53]. Interestingly, macrophages in perivascular adipose tissue infiltrate in response to vascular injury, inducing a BAT-like phenotype resembling cold- or β3-adrenergic receptor (β3AR)–induced WAT browning [54]. Further evidence indicates that adipose inflammation impairs adipocyte–sympathetic neuron crosstalk, thereby inhibiting BAT thermogenesis. Jiang et al. showed that inflammation-induced upregulation of metallothionein-2 in brown adipocytes reduces Zn secretion, which in turn suppresses activation of sympathetic neurons that normally release catecholamines to stimulate UCP1 expression and BAT thermogenesis [55]. Beyond sympathetic regulation, Wu et al. demonstrated that subcutaneous ATMs can directly promote WAT browning independent of sympathetic innervation. Mechanistically, anti-inflammatory macrophages repress Ets1 expression in adipocytes, thereby activating mitochondrial biogenesis, inhibiting mitochondrial clearance, and increasing mitochondrial content. Adipocyte-specific Ets1 knock-in mice are cold-intolerant, while Ets1-deficient mice exhibit enhanced energy expenditure and resistance to diet-induced metabolic disorders [56]. Notably, a recent study by Gonzalez-Hurtado et al. identified 13 distinct subsets of VAT-resident F4/80^+^ CD11b^+^ macrophages [57]. Among these, CD169^+^ CD11c^−^ adipose tissue macrophages were found to be enriched within a subpopulation of nerve-associated macrophages that declines with age. Depletion of these CD169^+^ nerve-associated macrophages in aged mice led to increased inflammaging and impaired lipolysis, indicating the development of catecholamine resistance in visceral adipose tissue. These studies indicate that macrophages play a vital role in regulating BAT burning by intervening the crosstalk between adipocytes and adipose sympathetic nerves.

## 3 Adipose inflammation and cancer therapy

Dysfunctional WAT fosters a tumor-permissive microenvironment through a synergistic interplay between adipocytes and ATMs that suppresses antitumor immunity. Adipocytes initiate this cascade by releasing inflammatory adipokines, nutrients (*e.g.*, palmitate), and hormones like leptin, which drive ATMs toward a pro-inflammatory phenotype. Crucially, these obesity-associated molecules induce PD-1 expression on TAMs and CD8^+^ T cells, thereby inhibiting their tumor killing capabilities. Concurrently, this inflammatory signaling promotes a "mechano-metabolic" shift where sATMs release TGFβ1 and TAMs generate collagen, resulting in a stiff, fibrotic extracellular matrix. This physical stiffness not only acts as a barrier to T-cell infiltration but also alters the metabolic landscape by depleting arginine, while simultaneously triggering biomechanical signaling that drives CD8^+^ T cells toward terminal exhaustion. This section will clarify how ATMs affects the functions of TAMs and tumor-resident CD8^+^ T cells.

### 3.1 WAT inflammation supports cancer progression

WAT has been increasingly recognized as a major contributor to cancer progression [[58], [59], [60]]. This occurs through the release of nutrients (*e.g.*, fatty acids, glutamine) [61,62] and inflammatory adipokines (*e.g.*, IL-6, leptin, adiponectin) that support cancer cell proliferation, invasion, and survival [[63], [64], [65]]. In addition, WAT-derived adipocytes generate a tumor-supportive microenvironment by reducing natural killer cell cytotoxicity, polarizing macrophages toward a pro-inflammatory phenotype, and promoting fibrosis and vascularization within tumors [[66], [67], [68], [69]]. Among these mechanisms, adipose inflammation, largely driven by WAT and recruited M1-type macrophages, plays a pivotal role in reshaping anti-tumor immunity and undermining the efficacy of cancer immunotherapy. Obesity-induced immune dysfunction has been demonstrated in both animal models and humans. Wang et al. reported that T cells from obese mice, rhesus macaques, and humans exhibit significantly elevated PD-1 expression and reduced proliferative capacity compared to non-obese controls [70]. Similarly, Bader et al. found that obesity not only increased PD-1 expression on CD8^+^ T cells in HFD–fed mice but also selectively upregulated PD-1 on tumor-associated macrophages (TAMs) [71]. Mechanistically, type I inflammatory cytokines and obesity-associated molecules—including IFN-γ, TNF, leptin, insulin, and palmitate—induced PD-1 expression on macrophages through mTORC1- and glycolysis-dependent pathways. PD-1 signaling then provided negative feedback to TAMs, suppressing glycolysis, phagocytosis, and antigen-presenting capacity. In contrast, PD-1 blockade enhanced macrophage glycolysis, increased CD86 and MHC class I/II expression, and improved T cell activation.

Besides, adipose inflammation may also affect tumor CD8^+^ T cells exhaustion by interfering with tumor extracellular matrix stiffness. As has mentioned above, septal ATMs support white adipocyte differentiation and inhibit thermogenic potential by locally releasing TGFβ1, a signal that also contributes to collagen secretion by tumor-associated macrophages. Tharp et al. showed that tumor-associated macrophages adapt to the rigid, fibrotic tumor microenvironment by activating a TGFβ-driven collagen-producing program that reshapes local metabolism [72]. In this state, macrophages deplete extracellular arginine while generating proline and releasing ornithine, creating nutrient conditions that weaken CD8^+^ T-cell activity in breast cancer. As a result, a stiff tumor matrix can hinder antitumor immunity not only by limiting T-cell infiltration but also by inducing a mechano-metabolic shift in macrophages that renders the microenvironment unfavorable for effective CD8^+^ T-cell responses and reduces the efficacy of immunotherapy. Increased tumor stiffness also causes CD8^+^ T-cell exhaustion. Zhang et al. identified the transcription factor Osr2 as a key integrator of biomechanical cues that drive terminal exhaustion in tumor-reactive CD8^+^ T cells [73]. Osr2 is preferentially upregulated in deeply exhausted, tumor-specific CD8^+^ T cells through the combined input of TCR activation and mechanical stress transmitted *via* the Piezo1–calcium–CREB pathway. Loss of Osr2 rejuvenates dysfunctional CD8^+^ T cells, including CAR-T cells, whereas enforced Osr2 expression accelerates their exhaustion in solid tumor settings. At the mechanistic level, Osr2 recruits HDAC3 to remodel chromatin in a manner that suppresses cytotoxic programs and reinforces the exhausted state. These findings position Osr2 as a biomechanical checkpoint whose inhibition may enhance antitumor immunity and improve immunotherapy outcomes. Collectively, these studies reveal a potential pathway that inflammatory adipose tissue may affect or support the progression of solid tumors.

The impact of weight loss on immune function has been increasingly recognized. Dyck et al. demonstrated that obesity accelerates tumor growth while impairing CD8^+^ T cell infiltration, proliferation, and effector function, largely due to disrupted chemokine expression and amino acid metabolism [74]. Notably, despite these suppressive effects, immunotherapy regimens using anti–PD-1 alone or in combination with a cancer vaccine induced tumor rejection in both lean and obese mice and partially restored pro-inflammatory CD8^+^ T cell activity. In humans, obesity was similarly associated with reduced CD8^+^ T cell infiltration in endometrial cancers, whereas weight loss following metabolic surgery promoted tumor regression and reversed this “cold” immune phenotype. Consistently, in a diet-induced obesity mouse model, Piening et al. showed that while both dietary restriction and semaglutide reduced body weight, only dietary restriction restored CD8^+^ T cell function and improved responses to immunotherapy [75]. While both interventions reduced body weight, only dietary restriction restored CD8^+^ T cell function and improved immunotherapy efficacy. In contrast, semaglutide failed to normalize T cell activity, underscoring the limitation of current AOMs, which primarily reduce adiposity without adequately resolving adipose inflammation [45]. Interestingly, tirzepatide, which exhibits partial anti-inflammatory activity, was reported to reduce tumor growth rates by ∼50 %, largely through lowering circulating leptin, a key driver of chronic low-grade inflammation [76]. The importance of targeting adipose inflammation is further highlighted by Zhao et al., who compared intermittent energy restriction and a low-fat diet, alone or in combination with paclitaxel, in a mouse model of obesity-associated endometrial cancer [77]. Both interventions reduced weight, reversed obesity-induced alterations in insulin, leptin, and inflammatory factors, and decreased tumor incidence and mass, with intermittent energy restriction producing the most favorable antitumor immune and metabolic environment. Collectively, these findings establish that obesity-associated adipose inflammation impairs both T cell and macrophage function, thereby reducing immunotherapy efficacy. Thus, development of next-generation anti-inflammatory AOMs may represent a pivotal strategy not only for treating obesity but also for enhancing cancer immunotherapy outcomes.

### 3.2 BAT thermogenesis inhibits cancer progression

**Fig. 3:**
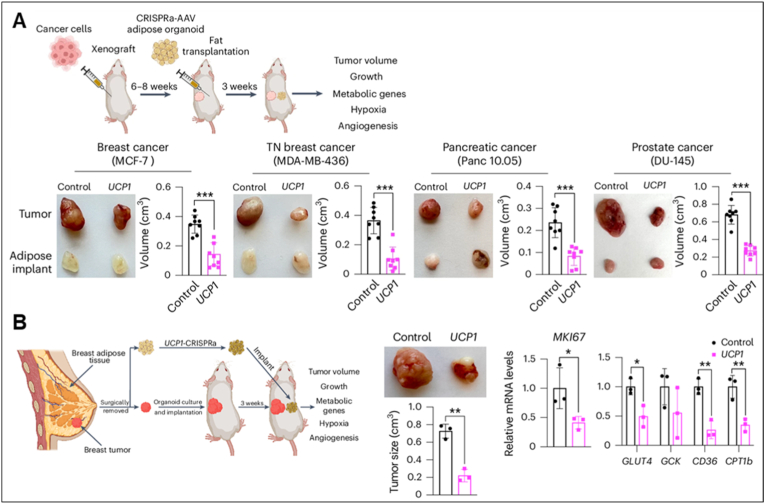
**Engineered BAT suppresses tumor progression.** (A) Co-transplantation of xenografts with UCP1-CRISPRa–treated human adipose organoids in immune-deficient SCID mice. (B) Co-transplantation of breast cancer organoids with UCP1-CRISPRa–treated breast adipocytes in immune-deficient SCID mice [89]. Reproduced with permission from the authors.

In contrast to WAT, BAT exhibits tumor-suppressive properties, though effects may vary by cancer type [78]. Glucose uptake is essential for both cancer glycolysis and non-shivering thermogenesis in adipose tissue [79,80]. Since most cancers rely heavily on glycolysis to fuel their rapid growth, invasion, and metastasis [81,82], activation of BAT thermogenic metabolism by cold exposure or pharmacological stimulation diverts blood glucose into adipocytes, limiting its availability to tumor cells [[83], [84], [85]]. For example, BAT overexpression of translationally controlled tumor protein enhances systemic metabolic homeostasis through UCP1-mediated thermogenesis [86]. UCP1-dependent proton leak is indispensable for thermogenesis and improves systemic glucose utilization, and recent studies demonstrate that BAT thermogenesis can suppress tumor growth by competing with cancer cells for glucose [87]. Supporting this, Seki et al. showed that cold exposure markedly inhibited the growth of multiple solid tumors, including highly aggressive cancers such as pancreatic carcinoma [88]. Mechanistically, cold-induced BAT thermogenesis reduced systemic glucose levels and impaired glycolysis in tumor cells. Importantly, BAT removal or feeding mice a high-glucose diet restored tumor growth under cold conditions, while genetic deletion of UCP1 abolished the anti-cancer effects of BAT thermogenesis. In a pilot human study, mild cold exposure substantially activated BAT in both healthy volunteers and a cancer patient, which corresponded with reduced tumor glucose uptake. In addition to cold-induced activation, BAT engineering strategies have been developed to exploit its anti-tumor potential. Nguyen et al. generated engineered adipocytes with enhanced UCP1 expression, which increased glucose and fatty acid uptake [89]. Placement of these engineered adipocytes near cancer cells or xenografts significantly suppressed tumor growth. Transplantation of engineered adipose organoids into pancreatic and breast cancer mouse models reduced tumor progression, angiogenesis, and hypoxia. Moreover, co-culture of patient-derived engineered adipocytes with breast cancer organoids markedly suppressed tumor cell proliferation (Fig. 3). Collectively, these studies highlight BAT thermogenesis and engineering as innovative strategies to restrict cancer growth through metabolic competition, providing a promising complement to conventional immunotherapies.

Conclusively, it is clearly that ATM plays a vital role in controlling WAT formation and browning, and BAT thermogenesis. Targeting.

## 4 Anti-inflammatory nanomedicines for obesity therapy

While targeting and reshaping dysfunctional ATMs in WAT represents a promising therapeutic strategy, it remains difficult to achieve with current interventions. Existing AOMs generally lack specific immunomodulatory functions, while potent preclinical anti-inflammatory agents are often hindered by poor bioavailability. Alternatively, tissue-targeted nanomedicine represents a promising next-generation strategy to overcome the limitations of conventional anti-obesity therapies. By improving drug solubility, controlling release kinetics, enhancing tissue-specific delivery, and reducing off-target toxicity, nanomedicine can significantly increase therapeutic efficacy while minimizing systemic side effects [90]. Although numerous nanomedicine-based interventions for obesity have been well summarized [91], their broader implications for cancer therapy remain underexplored. This section will therefore focus on nanomedicines with dual anti-obesity and anti-cancer potential, specifically those designed to reduce adipose tissue inflammation, reprogram pro-inflammatory immune cell phenotypes, promote WAT browning or BAT thermogenesis, and restore adipocyte–neuron crosstalk. By modulating these mechanisms, anti-obesity nanomedicines may not only facilitate sustainable weight loss but also enhance the effectiveness of cancer therapies, including immunotherapy and metabolic interventions.

### 4.1 Nanomedicines alleviate adipose inflammation

Adipose inflammation is a central driver of chronic low-grade systemic inflammation and metabolic dysfunction, while also impairing anti-tumor immune responses and promoting cancer progression. Lipid accumulation and tissue hypoxia caused by the rapid expansion of WAT provide a basic environment for the generation of adipose inflammation. Fatty acid binding proteins (FABPs) are small intracellular lipid-binding proteins that play a central role in free fatty acid (FFA) metabolism [92]. By facilitating lipid transport and storage across multiple cell types, FABPs are closely associated with metabolic disorders such as obesity and diabetes [93]. In particular, FABP4 (A-FABP) binds FFAs, promoting lipid accumulation and the release of FFAs and adipocytokines [94], and elevated FABP4 levels in adipocytes have been strongly linked to obesity [95,96]. To therapeutically target this pathway, Choi et al. developed a self-assembled oligopeptoplex system for adipocyte-specific shRNA delivery against FABP4/5. This nanoplatform achieved efficient gene silencing in adipose tissue and, in HFD-induced type 2 diabetes mouse model, significantly improved both obesity and insulin sensitivity [97]. Another hallmark of obese adipose tissue is its dependence on angiogenesis. WAT is highly vascularized, and its expansion requires extensive blood supply to address the formation of hypoxia. In obesity, WAT secretes pro-angiogenic factors, promoting blood vessel growth to sustain hypertrophic adipocytes with oxygen and nutrients [98]. Targeting this vascular network has therefore emerged as an attractive anti-obesity strategy. Xian et al. developed an oral nanodrug system consisting of zein-core nanoparticles decorated with adipose-homing peptides to enhance the delivery of celastrol (Cel) [99]. This nanoplatform significantly improved the oral bioavailability of Cel and exerted potent anti-angiogenic effects in WAT, thereby suppressing diet-induced obesity.

**Fig. 4:**
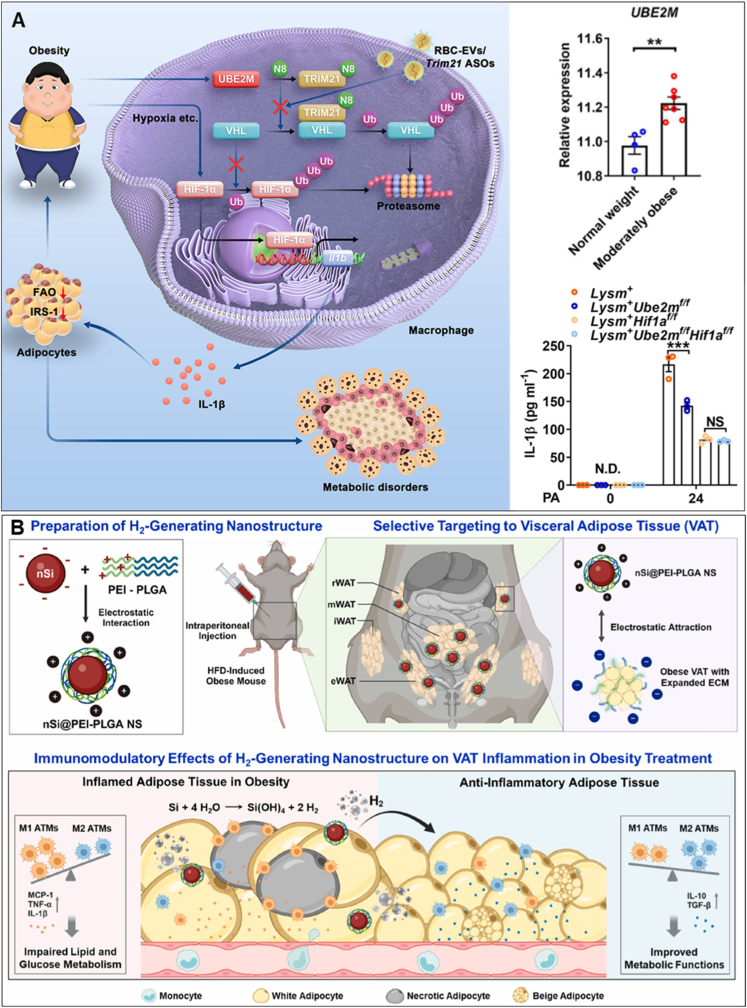
**Nanomedicine directly reduces adipose inflammation.** (A) *Trim21* antisense oligonucleotide–loaded red blood cell extracellular vesicles suppress adipose tissue macrophage–mediated inflammation [108]. (B) H_2_-generating nanomedicine mitigates obesity-induced adipose inflammation by reprogramming macrophages [109]. (A) was reproduced with permission from 2023 Elsevier Inc., (B) was reproduced with permission from 2026 Elsevier Inc.

Moreover, a simple strategy to address adipose inflammation is by supplementing anti-inflammatory cytokines into adipose tissues; for example, IL-10–conjugated liposomes significantly suppressed adipose inflammation [100]. However, since the adipose inflammatory microenvironment is orchestrated by diverse biomolecules from multiple cell types, more effective approaches focus on directly reprogramming adipose tissue macrophages—the key mediators of obesity-induced inflammation through secretion of TNF-α, IL-6, and other pro-inflammatory cytokines. Several nanomedicines have been developed to suppress pro-inflammatory pathways and stimulate endogenous pro-resolving mechanisms [101,102]. Sharma et al. designed oleic acid–grafted chitosan nanomicelles conjugated with α-D-mannopyranosylphenyl isothiocyanate or adipose-homing peptide (CKGGRAKDC) to selectively target adipose tissue macrophages or adipocytes *via* GLUT1-and prohibitin receptor–mediated uptake [103]. By delivering shRNA against TNF-α and MCP-1 into obese-diabetic mice, this system significantly reduced pro-inflammatory cytokines (TNF-α, MCP-1, IL-6, IL-1β) and increased adiponectin levels. To enhance adipose tissue macrophage specificity, Yong et al. developed an oligopeptide (ATS-9R)–based non-viral delivery system that selectively targets VAT macrophages [104]. When loaded with TNF-α–converting enzyme shRNA (ATS-9R/shTACE) and administered intraperitoneally, this nanomedicine markedly reduced VAT inflammation and improved type 2 diabetes outcomes. Similarly, Wang et al. identified CCL2 as a pro-inflammatory driver enriched in macrophages from gestational diabetes mellitus patients and HFD-fed mice [105]. Silencing CCL2 with siRNA blocked calcium transfer between endoplasmic reticulum and mitochondria, reduced ROS generation, and alleviated VAT inflammation and insulin resistance. Moreover, macrophage-targeted polysaccharide nanomedicines have also shown promise. Ma et al. synthesized dextran conjugates with tunable size, which selectively accumulated in VAT macrophages after peritoneal administration, achieving >2-fold higher concentrations in adipose tissue than in liver [106]. This strategy enabled combined imaging, drug delivery, and phenotypic reprogramming of macrophages, resulting in significant reductions in VAT inflammation. Building on this, Prabhu et al. demonstrated that dextran–nanopolysaccharide delivery of glucocorticoids produced nearly 20-fold higher potency than free drug, resulting in robust fat mobilization, weight loss, improved glucose tolerance, and durable suppression of pro-inflammatory gene expression even weeks after treatment cessation [107]. Beyond cytokine suppression, recent studies have targeted upstream molecular pathways. Lu et al. found that macrophage UBE2M promoted TRIM21-mediated degradation of the VHL tumor suppressor, stabilizing HIF-1α and driving IL-1β–dependent inflammation (Fig. 4A) [108]. Delivery of *Trim21* antisense oligonucleotides *via* red blood cell–derived extracellular vesicles effectively blocked this axis, ameliorating obesity-induced inflammation and related metabolic disorders.

Gas therapy also contributes to modulate the adipose microenvironment. In the context of obesity, chronic low-grade inflammation can impair insulin signaling, leading to insulin resistance. H_2_ molecules are safe and effective anti-inflammatory agents. Pham et al. developed a nanosilicon-based hydrogen (H_2_)–generating nanostructure with a positively charged coating that preferentially accumulated in the negatively charged extracellular matrix of obese VAT (Fig. 4B) [109]. Sustained H_2_ release reprogrammed adipose tissue macrophages by restoring the M1/M2 balance, thereby reducing VAT inflammation, improving adipocyte function, and enhancing systemic metabolic health. Similarly, Liu et al. designed a Zn–Fe primary-battery micro/nanostructure capable of controlled H_2_ generation synchronized with gastric emptying to maximize H_2_ bioavailability and therapeutic efficacy [110]. By tuning the Zn/Fe ratio to 1:100, the nanostructure achieved a 3-h H_2_ generation period—matching the gastric emptying time in mice. Oral administration of Zn–Fe micro/nanostructures (200 mg/kg) in leptin-deficient (ob/ob) mice resulted in sustained H_2_ accumulation in the liver, adipose tissue, and skeletal muscle, leading to significant improvement in insulin resistance and systemic inflammation without observable toxicity. This innovative approach provides an efficient and safe strategy to enhance the therapeutic outcomes of hydrogen-based gas therapy for obesity-associated metabolic disorders.

**Fig. 5:**
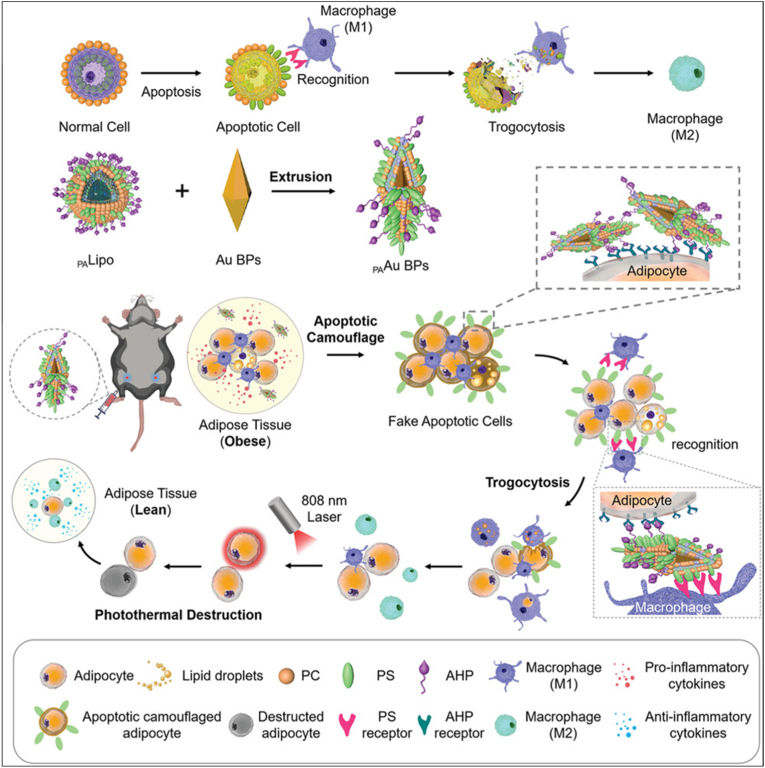
**Photothermal responsive nanomedicine for reshaping macrophage anti-inflammatory polarization.** During apoptosis, externalization of PS on the cell membrane facilitates macrophage recruitment and clearance of apoptotic cells through a trogocytosis process. Regulation of adipocyte clearance by PAAu BPs, which camouflage adipocytes with apoptotic features through extracellular embedment, thereby promoting macrophage-mediated phagocytosis and immune remodeling of adipose tissue [116]. Reproduced with permission from 2022 Wiley – VCH GmbH.

Besides, numerous nanomaterials exhibit anti-oxidant functions to scavenge adipose ROS. For instance, Ding et al. engineered aptamer-modified gold nanoclusters (Apt-Au25 NCs) with strong catalase- and superoxide dismutase–like activities [111]. These nanozymes efficiently scavenged ROS in WAT adipocytes, suppressed oxidative stress, and attenuated adipose inflammation with minimal cytotoxicity. With the advancement of nanomaterials, various gold and silver (Au and Ag) nanostructures have been explored for photothermal therapy (PTT) in obesity due to their strong localized surface plasmon resonance effect, which enables efficient heat generation for targeted tissue ablation [112]. Recently, Au nanoparticles have been utilized to induce lipolysis of lipid droplets and adipocyte necrosis *via* photothermal heating [113]. Moreover, macrophage-mediated immune clearance has emerged as a feasible immunotherapeutic approach for obesity by promoting the removal of adipocytes. Since macrophages recognize apoptotic cells *via* surface “eat-me” signals such as phosphatidylserine (PS) [114,115], PS-camouflaged adipocytes can be selectively cleared to prevent weight rebound. Yan et al. the first immunotherapy strategy for obesity by inducing apoptotic camouflage of adipocytes using gold nanobipyramids (PAAu BPs) engineered with adipose-targeting and apoptotic cell–mimicking properties (Fig. 5) [116]. This design activates macrophage-mediated clearance of adipocytes while reprogramming macrophages from a pro-inflammatory M1 to an anti-inflammatory M2 phenotype, thereby reshaping the adipose immune microenvironment and preventing weight regain. Following inguinal injection, PAAu BPs reduced body weight in obese mice by 24.4 %, and when combined with photothermal lipolysis, achieved up to a 33.3 % reduction with minimal rebound. Collectively, these studies demonstrate that nanomedicine-enabled WAT browning strategies not only reduce adiposity and metabolic dysfunction but may also create a metabolically favorable, anti-inflammatory microenvironment with potential implications for enhancing cancer therapy.

Summarily, these findings demonstrate that nanomedicine-based strategies targeting adipose tissue macrophages, inflammatory mediators, or the adipose microenvironment hold significant promise for reducing obesity-induced inflammation, with important implications for both metabolic disease management and cancer therapy.

### 4.2 Nanomedicines promote WAT browning and BAT burning

BAT plays a central role in systemic energy expenditure and glucose homeostasis, and its unique thermogenic capacity makes it an attractive therapeutic target for obesity and its metabolic complications [117]. Converting WAT into thermogenic BAT represents a promising strategy for obesity management and its associated pathologies [118]. Multiple WAT browning agents—including acetate, capsaicin, resveratrol, berberine, thiazolidinediones, leptin, and various microRNAs—have been identified [119], with natural compounds drawing particular attention due to their safety and reduced side effects [120]. For instance, resveratrol enhances mitochondrial biogenesis and UCP1 expression *via* AMPK and peroxisome proliferator-activated receptor γ (PPARγ) activation [121,122]. Encapsulation of resveratrol into biocompatible, ASC-targeted nanoparticles improved solubility, stability, and selectively induced beige adipogenesis, resulting in weight reduction and metabolic improvement in obese models [123]. Similarly, turmeric-derived nanovesicles (Rec-tNVs) efficiently loaded with curcumin promoted WAT browning through PPARγ and UCP1 upregulation [124,125]. Other phytochemicals such as emodin, quercetin, tangeretin, and lactucin have also been linked to WAT browning [[126], [127], [128], [129]], and their integration with nanotechnology may further potentiate therapeutic efficacy. To enhance therapeutic precision, novel delivery strategies have been developed. Zhang et al. designed a degradable microneedle patch loaded with rosiglitazone, glucose oxidase, and catalase for local WAT browning [130]. In obese mouse models, treatment induced WAT browning of visceral white fat pads within six days, accompanied by improved systemic metabolism, indicating a sustained effect of the WAT browning agents. After four weeks, diet-induced obese mice exhibited a 30 % reduction in visceral WAT, enhanced energy expenditure, increased fatty acid oxidation, improved body weight control, and greater insulin sensitivity.

**Fig. 6:**
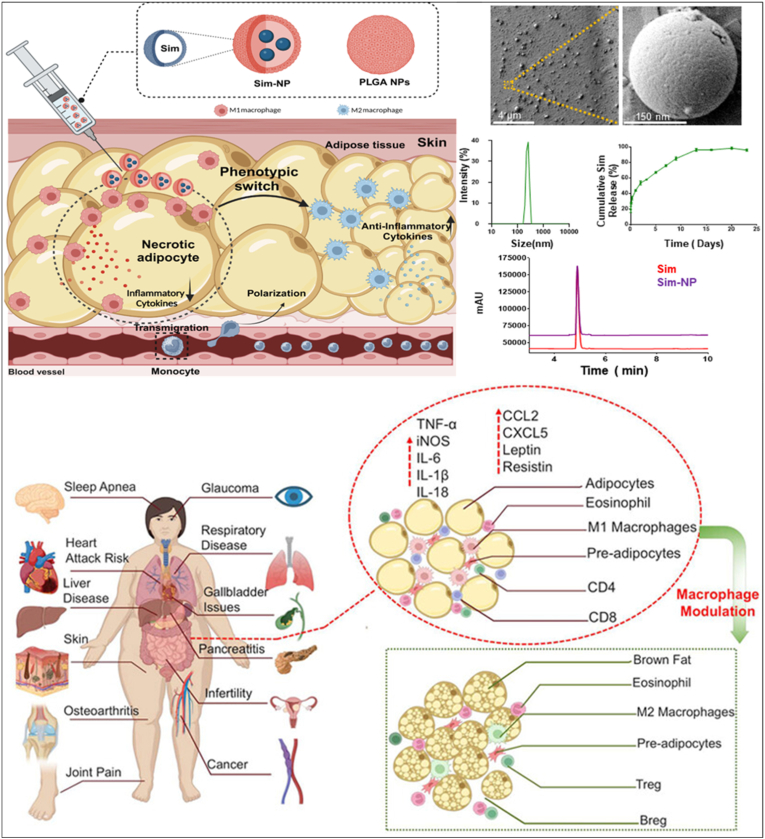
**Local delivery of nanomedicine promotes WAT browning.** The nanomedicine gradually releases simvastatin from the polymeric PLGA matrix, skewing macrophage polarization from the pro-inflammatory M1 phenotype toward the anti-inflammatory M2 phenotype, thereby facilitating the conversion of white fat to brown fat. In WAT, this nanoplatform modulates adipose function by reducing macrophage-mediated inflammation and cytokine secretion [132]. Reproduced with permission from 2024 American Chemical Society.

Recent work highlights the role of immune modulation in WAT browning. Applegate et al. reported a dextran-based nanomedicine delivering PPARα/γ agonists to adipose tissue macrophages, resulting in weight loss, restored glucose tolerance, and WAT browning across multiple obesity models [131]. Notably, within 1 week of treatment, adipose tissue macrophages declined and became lipid-laden, with a concurrent reduction in lipid-rich extracellular vesicles. By 2 weeks, glucose tolerance normalized, accompanied by weight loss and reduced food intake, and by 4 weeks, WAT browning and improved hepatic steatosis were evident. These effects were reproducible across multiple rodent obesity models and were significantly greater than those achieved with equivalent doses of free drugs, highlighting the advantage of targeted PPAR activation in ATMs. Moreover, Mohaghegh et al. demonstrated that local administration of simvastatin-encapsulated poly(lactic-co-glycolic acid) (PLGA) nanoparticles effectively modulated adipose tissue macrophage polarization, reducing the M1/M2 ratio and shifting the local immune microenvironment toward an anti-inflammatory state to mitigate lipid accumulation and combat obesity (Fig. 6) [132]. In HFD-induced obese mice, this localized nanomedicine enabled the controlled release of simvastatin within adipose tissue, directly influencing macrophage activity, promoting WAT browning, and inducing significant weight loss.

Nitric oxide (NO) can also enhance metabolic rate by promoting mitochondrial biogenesis and activating BAT. Ren et al. developed a peptide amphiphile–NO-releasing nanomatrix gel (PANO gel) as a novel approach to deliver exogenous NO, thereby improving metabolic rate and cognitive function through BAT activation [133]. In HFD–induced obese mouse model, subcutaneous injection of PANO gel into the BAT region every two weeks for 12 weeks significantly reduced body weight gain, improved glucose tolerance, and lowered fasting serum insulin and leptin levels compared with controls. PANO gel treatment enhanced insulin signaling in muscle, liver, and epididymal WAT, while reducing inflammation, promoting lipolysis, and decreasing circulating lipids and hepatic triglycerides. Notably, PANO gel increased UCP1 expression in brown and beige fat depots, elevated cerebral blood flow, and improved learning and memory performance, highlighting its potential as a nanomedicine to combat obesity and related metabolic and cognitive dysfunctions.

**Fig. 7:**
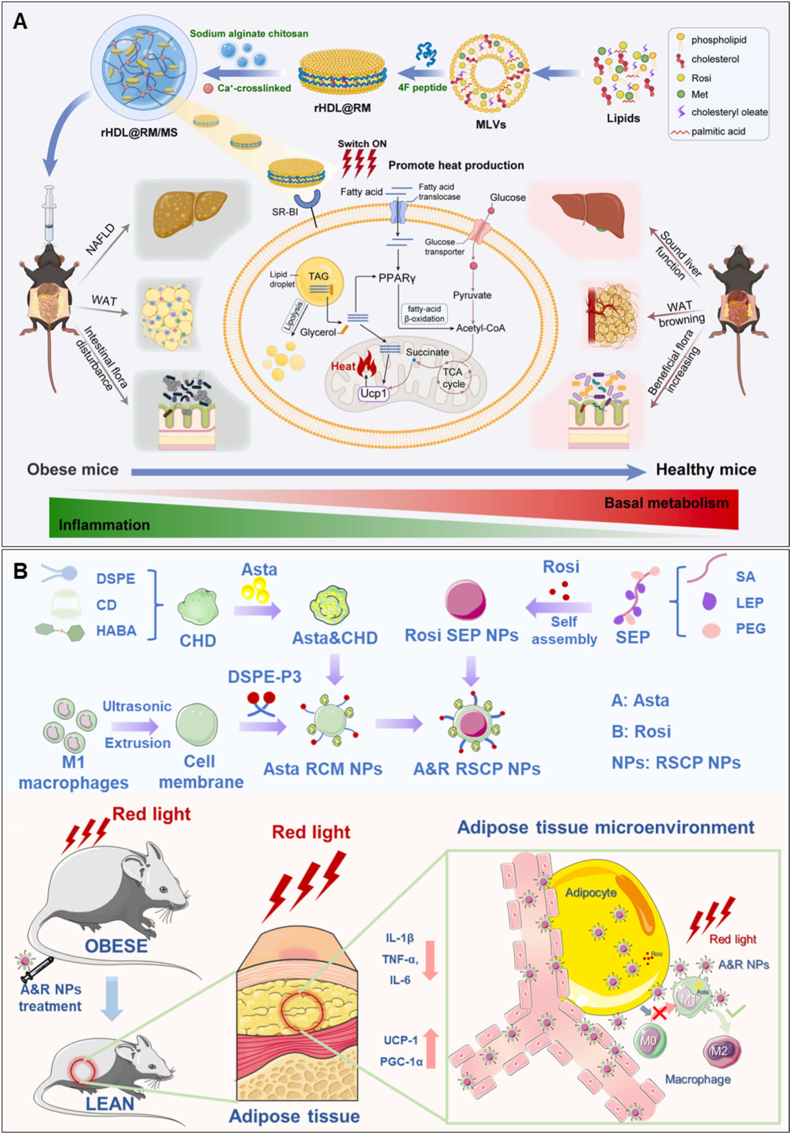
**Biomimetic nanomedicines for promoting WAT browning.** (A) Adipose tissue–targeted sequential biomimetic nanomedicine regulates glycolipid metabolism for systemic obesity therapy. After being swallowed, the composite microspheres swell inside the gut to release their cargo, allowing rHDL@RM to enter the circulation. The particles then seek out SR-BI on adipocytes to transport RM inside, stimulating WAT browning and enhancing energy consumption through heat generation. Additionally, the therapy acts on the liver and intestine to alleviate NAFLD, aiding in hepatic recovery and increasing the population of helpful intestinal microbes [135]. (B) Photosensitive bionic delivery platform that can improve WAT low-grade chronic inflammation and promote WAT browning to normalize metabolic disorders and reducing obesity and insulin resistance [139]. (A) was reproduced with permission from 2024 Elsevier Ltd. (B) was reproduced with permission from 2025 Wiley – VCH GmbH.

Photothermal therapy has also been utilized for promoting WAT browning. Zan et al. engineered a thermos-responsive hydrogel containing copper sulfide nanodots for mild photothermal remodeling of subcutaneous WAT, achieving systemic metabolic benefits and prevention of HFD-induced obesity [134]. Impressively, the subsequent photothermal-pharmacotherapy completely inhibited obesity development in HFD–fed mice. Beyond inducing local SAT remodeling and mass reduction at the treatment site, it also produced systemic metabolic benefits, including reduced VAT mass and improved whole-body metabolism. Similarly, Chen et al. constructed biomimetic discoidal recombinant HDL (rHDL) nanoparticles co-delivering rosiglitazone and metformin (rHDL@RM) to WAT, liver, and intestine *via* scavenger receptor class B type I targeting (Fig. 7A) [135]. This system promoted WAT browning, mitochondrial biogenesis, and thermogenesis, while effectively reducing obesity comorbidities including NAFLD, dyslipidemia, gut dysbiosis, and systemic inflammation in obese mice. Additionally, prussian blue nanoparticles (PBNPs), serving as dual-function photothermal agents and nanozymes with ROS-scavenging and oxygen-generating activities [136], were encapsulated within a biocompatible silk fibroin hydrogel for transdermal administration into [137]. In 3T3-L1 adipocytes, mild photothermal (808 nm, 0.4 W/cm^2^, 5 min) and nanocatalytic therapy effectively induced WAT browning, enhanced lipolysis, and mitigated oxidative stress. In obese mouse models, this synergistic treatment achieved a pronounced reduction in subcutaneous WAT mass (53.95 %) and visceral fat (65.37 %), accompanied by a 9.78 % decrease in body weight, alleviation of hyperlipidemia and systemic inflammation, and complete reversal of type 2 diabetes. Likewise, Mohaghegh et al. demonstrated that apigenin-loaded PLGA nanoparticles promoted M2 macrophage polarization, alleviated inflammation, and enhanced WAT browning in HFD-fed mice [138]. More recently, Song et al. developed red light-responsive biomimetic nanoparticles (RSCP) for dual-targeted delivery of astaxanthin and rosiglitazone to pro-inflammatory macrophages and white adipocytes, respectively (Fig. 7B) [139]. These findings demonstrate that the dual-targeting, multi-signal-responsive RSCP system—sensitive to red light and pH variations—precisely delivers astaxanthin (Asta) to M1-like macrophages and Rosi to white adipocytes, effectively reducing WAT inflammation and promoting WAT browning. Furthermore, RSCP-mediated Asta and Rosi treatment significantly improved insulin resistance and ameliorated metabolic disorders in both HFD-induced and genetically obese mouse models.

Collectively, these advances demonstrate that nanomedicine-based strategies can effectively reprogram adipose tissue metabolism by promoting WAT browning and enhancing BAT thermogenesis. Through precise delivery of bioactive compounds, immune modulators, and gasotransmitters, these platforms not only improve local adipose remodeling but also restore systemic metabolic homeostasis, alleviate inflammation, and reverse obesity-related comorbidities. Integrating photothermal, catalytic, and immunomodulatory mechanisms further amplifies therapeutic outcomes, establishing nanomedicine as a versatile and powerful tool for combating obesity and its metabolic complications. Nevertheless, several critical limitations in the current landscape must be acknowledged to guide future translational efforts. First, the predominant reliance on small-cohort murine models often fails to capture the complexity of human pathophysiology, while the prevalence of acute or single-dose treatment paradigms limits insights into the safety and efficacy of chronic administration required for obesity management. Furthermore, rigorous evaluations of long-term biodistribution and potential toxicity are frequently absent. Finally, there remains an insufficient examination of sex differences, a crucial variable given the distinct biological mechanisms governing adipose tissue metabolism and remodeling in males versus females.

## 5 Perspectives

As the global prevalence of obesity and cancer continues to rise, the complex and bidirectional relationship between these two diseases has garnered increasing attention [140,141]. Chronic low-grade inflammation originated from adipose inflammation not only contributes to metabolic dysfunction but also fosters a tumor-promoting microenvironment through the secretion of cytokines, adipokines, and growth factors [142,143]. Despite the remarkable progress of AOMs such as GLP-1 receptor agonists, their therapeutic benefits largely stem from appetite suppression and energy intake reduction, without directly addressing the pathological features of adipose inflammation and immune dysregulation. Consequently, current AOM-based weight reduction strategies fail to fully restore adipose immune homeostasis or reverse the compromised anti-tumor immune surveillance [75]. Considering the role of adipose inflammation and its correlation with cancer progression, next-generation AOMs may benefit from anti-inflammatory compounds or nanomaterials that directly eliminated adipose inflammation or promote BAT metabolism, as well as metal ions that serve as immunomodulator to interfere with immune cell function and communicator between cells.

### 5.1 Dual functional natural compounds

Natural bioactive compounds derived from edible plants and traditional Chinese medicines have long been recognized as valuable sources for drug discovery and development [144]. Many of these compounds possess potent antioxidant and anti-inflammatory properties [145]. Among them, resveratrol, curcumin, quercetin, vitamin E, alpha-lipoic acid, vitamin C, chlorogenic acid, lycopene, fucoxanthin, and berberine rank among the top natural agents with well-established anti-inflammatory efficacy [146]. For example, resveratrol exerts broad anti-inflammatory actions by suppressing nuclear factor kappa-light-chain-enhancer of activated B (NF-κB) signaling and other pro-inflammatory pathways, leading to reduced cytokine release and improved immune homeostasis [147]. It has been extensively investigated for treating a range of inflammation-related disorders, including metabolic and cardiovascular diseases [148]. Similarly, curcumin, the principal bioactive component of turmeric, inhibits key inflammatory mediators such as cyclooxygenase-2 and NF-κB, demonstrating significant benefits in alleviating chronic inflammatory conditions such as osteoarthritis and rheumatoid arthritis [149]. Other naturally occurring molecules—such as rutin, naringin, and α-tocopherol—have also been widely employed for inflammation control and oxidative stress mitigation [[150], [151], [152], [153], [154]]. Increasing evidence now supports their potential in obesity management, where chronic adipose inflammation plays a pathogenic role [155].

Beyond their anti-inflammatory actions, what distinguishes these compounds is their unique ability to promote WAT browning and BAT activation, thereby enhancing energy expenditure. Rutin, for instance, markedly upregulates AMPK signaling and UCP1 expression in both WAT and BAT, leading to significant reductions in weight gain, plasma lipid levels, and adipocyte hypertrophy in HFD–fed mice [156]. Curcumin has been shown to elevate thermogenic gene expression and mitochondrial biogenesis in inguinal WAT while upregulating β_3_-adrenergic receptor expression and circulating norepinephrine levels, both of which drive WAT browning [157]. Additional studies confirm that curcumin stimulates UCP1 expression through both PPAR-dependent and PPAR-independent mechanisms [158]. Likewise, naringin [159,160], resveratrol [161,162], capsaicin [163,164] and chlorogenic acid [165,166] have each been demonstrated to induce thermogenic reprogramming of adipocytes through UCP1 activation.

Importantly, most of these WAT browning-promoting compounds are naturally occurring dietary supplements with excellent biosafety profiles, enabling their long-term use for metabolic health improvement. Moreover, many of them also exhibit direct anti-tumor activities, including inhibition of cancer cell proliferation, angiogenesis, and metastasis, as well as enhancement of anti-tumor immune responses [167,168]. Collectively, these dual-functional compounds offer a compelling foundation for the development of next-generation AOMs. Such natural-compound-based AOMs not only alleviate adipose inflammation but also stimulate WAT browning and BAT thermogenesis, thereby restoring metabolic balance while potentially synergizing with cancer immunotherapy.

### 5.2 Drug-free nanomaterials

As discussed above, the most widely recognized role of nanomaterials in anti-obesity therapy is to deliver therapeutic molecules. However, emerging evidence has revealed that certain nanomaterials themselves possess intrinsic anti-inflammatory and immunomodulatory activities, demonstrating therapeutic potential across multiple diseases, particularly in cancer immunotherapy [[169], [170], [171]]. Leveraging their inherent physicochemical properties, these nanomaterials can effectively reprogram immune cell function within the tumor microenvironment [[172], [173], [174]], correcting aberrant conditions such as acidosis [[175], [176], [177]], hypoxia [178,179], and metal ion dysregulation [[180], [181], [182], [183]]. Similarly, adipose tissue in obesity also exhibits hypoxia [184], metal ion dysregulation (notably zinc deficiency) [185], and excessive ROS accumulation [186], all of which contribute to chronic adipose inflammation and impaired WAT/BAT metabolism.

To address tissue hypoxia, various nanozymes have been designed to convert endogenous ROS into oxygen, thereby simultaneously alleviating oxidative stress and oxygen deficiency. For example, Sun et al. developed acid-sensitive nanozymes based on peroxidized layered double hydroxide nanoparticles for self-supplied oxygen generation and lactate depletion [187]. By co-loading catalase and lactate oxidase, these nanozymes catalyzed sequential reactions—hydrolysis of MgO_2_ to generate H_2_O_2_, conversion of H_2_O_2_ into O_2_, and lactate oxidation—under acidic conditions, effectively relieving hypoxia and lowering lactate accumulation. In a subsequent study, a manganese (Mn)-layered double hydroxide nanoplatform was engineered as a defect-rich sono-chemo sensitizer to amplify ultrasound-triggered ROS generation for combined sono/chemodynamic therapy [188]. This platform not only enhanced singlet oxygen production by catalyzing endogenous H_2_O_2_ into O_2_ but also released Mn^2+^ that activated immune responses and reprogrammed the immunosuppressive tumor microenvironment. Similarly, Yang et al. designed manganese ferrite nanoparticle (MFN)-based nanovesicles grafted with hypoxia-responsive polymers. Under hypoxic conditions, these nanovesicles disassembled into individual MFNs, decomposing H_2_O_2_ to generate O_2_ for hypoxia relief [189].

Metal ions also play central roles in immune regulation, and metallodrugs have long been recognized for their therapeutic efficacy across multiple pathologies [190]. In obesity, zinc deficiency is a key metabolic abnormality that impairs BAT thermogenesis by disrupting adipocyte–sympathetic nerve crosstalk [55,191]. Zinc supplementation has been shown to facilitate weight loss in both humans and mice [[192], [193], [194]]. Recently, zinc-based nanomaterials have been widely explored in cancer immunotherapy due to their ability to restore intracellular and extracellular zinc balance. For instance, Cen et al. developed ZnS@albumin nanoparticles that triggered immunogenic cell death by activating the cyclic guanosine monophosphate-adenosine monophosphate synthase/interferon gene stimulator pathway and enhancing innate immunity [195]. Zeng et al. further demonstrated that ZnO nanoparticles selectively accumulated in papillary renal tumors, promoting dendritic cell recruitment and cytotoxic CD8^+^ T cell infiltration [196]. Zhao et al. also reported zinc-containing nano-aluminum adjuvants that enhanced dendritic cell autophagy and antigen presentation [197]. Intriguingly, Zhang et al. found that subcutaneous administration of ZnCl_2_ or zinc-doped nano-aluminum adjuvants not only inhibited tumor progression and metastasis but also prevented weight gain in lean tumor-bearing mice [196], suggesting potential translational value for obesity-associated cancer therapy. Beyond zinc-based systems, other nanomaterials also exhibit inherent anti-inflammatory effects [198]. For example, magnesium–aluminum layered double hydroxides promote macrophage M2 polarization through magnesium ion signaling in inflammatory diseases [153,199]. Likewise, a broad class of ROS-scavenging nanozymes has been shown to shift macrophage phenotypes from pro-inflammatory M1 to anti-inflammatory M2 states, effectively modulating inflammatory microenvironments [200]. Collectively, these studies highlight the promise of drug-free nanomaterials as multifunctional platforms capable of correcting metabolic and immune dysregulation in obesity.

### 5.3 Clinical translation and challenges

Despite the promising therapeutic potential of adipose-targeted strategies, several key barriers must be overcome before these approaches can be successfully translated into clinical practice. A central challenge lies in achieving precise and depot-specific biodistribution. Nanocarriers must maintain hemodynamic stability in circulation, resisting protein corona formation and premature drug leakage—both of which can alter pharmacokinetics and redirect nanoparticles away from adipose tissue. Even when circulation stability is optimized, deposition into adipose tissue remains inconsistent due to heterogeneous vascularization among adipose depots. Visceral fat, subcutaneous fat, and obesity-induced hypertrophic adipocytes exhibit markedly different perfusion characteristics. Unlike the relatively predictable enhanced permeability and retention effect observed in tumors, the poor vascular density and hypoxic microenvironment of inflamed adipose tissue often limit nanoparticle accumulation, resulting in variable dose–response relationships and complicating therapeutic standardization. Besides, immune compatibility represents a second major obstacle [201]. Although several inorganic and metal-based nanoplatforms offer strong catalytic or anti-inflammatory activity, they may also induce immunogenicity, including hypersensitivity responses or the formation of neutralizing antibodies during repeated dosing. These immune-mediated events not only raise safety concerns but may also diminish therapeutic efficacy over time, emphasizing the need for systematic immunotoxicity profiling during early development.

From a pharmaceutical standpoint, formulation stability and storage robustness further constrain clinical translation [202,203]. Many natural compounds—such as curcumin, resveratrol, and other plant-derived antioxidants—are chemically unstable and readily degraded by light exposure, oxidation, or hydrolysis. Ensuring adequate shelf-life requires advanced encapsulation methods and stringent storage conditions to preserve bioactivity [204,205]. Likewise, multi-component nanocarriers incorporating lipids, polymers, targeting ligands, or enzymes must maintain physicochemical stability over prolonged periods, which can be difficult to guarantee outside controlled laboratory settings. Producing multifunctional nanomedicines with reproducible size, charge, morphology, and drug loading efficiency requires sophisticated engineering controls and rigorous quality standards. Batch-to-batch variability remains a nontrivial hurdle, and the absence of well-defined regulatory frameworks for nanotherapeutics that act simultaneously as metabolic modulators and cancer immunotherapies complicates the approval pathway [206]. Establishing standardized characterization, safety assessment, and GMP-grade production pipelines will be essential for advancing these technologies toward clinical trials.

Finally, while thermogenic reprogramming strategies hold significant therapeutic promise, their physiological risks must be carefully considered. Systemic activation of thermogenic pathways can impose cardiovascular strain, potentially inducing tachycardia, hypertension, or increased myocardial workload [207,208]. In parallel, aggressive induction of lipolysis risks elevating circulating free fatty acid levels to a point that promotes systemic lipotoxicity or ectopic fat deposition in vital organs such as the liver and heart [209]. Fat depot-specific heterogeneity adds further complexity, as visceral and subcutaneous adipocytes respond differently to browning stimuli, making uniform remodeling difficult to achieve in patients [210]. Therefore, careful dose optimization and real-time metabolic monitoring are necessary to define a safe therapeutic window that enhances energy expenditure without triggering adverse effects such as uncontrolled hyperthermia, excessive catabolism, or cachexia-like wasting.

## 6 Summary

This review summarizes the impact of adipose inflammation on both anti-obesity and cancer therapies. By elucidating the underlying mechanisms and current AOMs that target adipose inflammation, we identify two major frontiers for next-generation AOM development: (1) natural compounds, widely used as nutraceuticals, that alleviate adipose inflammation while promoting WAT browning; and (2) drug-free nanomaterials, extensively studied in tumor immunotherapy, that harness intrinsic physicochemical properties to remodel the tissue microenvironment, modulate immune responses, and promote BAT activity and thermogenesis. Since most clinically available weight-loss agents lack potent anti-inflammatory properties and therefore fail to enhance cancer treatment efficacy, our review provides new insights into designing innovative obesity therapeutics that may concurrently benefit metabolic health and tumor immunotherapy outcomes.

## CRediT authorship contribution statement

**Fang Zhou:** Writing – original draft, Funding acquisition. **Xiaofang Li:** Writing – review & editing. **Jianping Jiang:** Writing – review & editing, Funding acquisition. **Lingxiao Zhang:** Writing – review & editing, Visualization, Funding acquisition, Conceptualization.

## Declaration of competing interest

The authors declare that they have no known competing financial interests or personal relationships that could have appeared to influence the work reported in this paper.

## Data Availability

No data was used for the research described in the article.
